# Microbiota profiles in pre-school children with respiratory infections: Modifications induced by the oral bacterial lysate OM-85

**DOI:** 10.3389/fcimb.2022.789436

**Published:** 2022-08-16

**Authors:** Susanna Esposito, Stefania Ballarini, Alberto Argentiero, Luca Ruggiero, Giovanni A. Rossi, Nicola Principi

**Affiliations:** ^1^ Pediatric Clinic, Department of Medicine and Surgery, University of Parma, Parma, Italy; ^2^ Medicine and Surgery Department, University of Perugia, Perugia, Italy; ^3^ Fondazione Istituti di Ricovero e Cura a Carattere Scientifico Cà Granda Ospedale Maggiore Policlinico, Milan, Italy; ^4^ Department of Pediatrics, Unit of Pediatrics Pulmonology and Respiratory Endoscopy, G. Gaslini University Hospital, Genoa, Italy; ^5^ Professor Emeritus of Pediatrics, Università degli Studi di Milano, Milan, Italy

**Keywords:** children, microbiota, respiratory infection, dysbiosis, bacterial lysates, OM-85

## Abstract

To describe microbiota profiles considering potential influencing factors in pre-school children with recurrent respiratory tract infections (rRTIs) and to evaluate microbiota changes associated with oral bacterial lysate OM-85 treatment, we analyzed gut and nasopharynx (NP) microbiota composition in patients included in the OM-85-pediatric rRTIs (OMPeR) clinical trial (https://www.clinicaltrialsregister.eu/ctr-search/trial/2016-002705-19/IT). Relative percentage abundance was used to describe microbiota profiles in all the available biological specimens, grouped by age, atopy, and rRTIs both at inclusion (T0) and at the end of the study, after treatment with OM-85 or placebo (T1). At T0, *Firmicutes* and *Bacteriodetes* were the predominant genera in gut and *Proteobacteria*, *Firmicutes*, and *Actinobacteria* were the predominant genera in NP samples. Gut microbiota relative composition differed with age (<2 vs. ≥2 years) for *Firmicutes, Proteobacteria, Actinobacteria* (phyla) and *Bifidobacterium, Ruminococcus, Lachnospiraceae* (genera) (*p* < 0.05). *Moraxella* was more enriched in the NP of patients with a history of up to three RTIs. Intra-group changes in relative percentage abundance were described only for patients with gut and NP microbiota analysis available at both T0 and T1 for each study arm. In this preliminary analysis, the gut microbiota seemed more stable over the 6-month study in the OM-85 group, whose mean age was lower, as compared to the placebo group (*p* = 0.004). In this latter group, the relative abundance of *Bacteroides* decreased significantly in children ≥2 years. Some longitudinal significant differences in genera relative abundance were also detected in children of ≥2 years for NP *Actinobacteria*, *Haemophilus*, and *Corynebacterium* in the placebo group only. Due to the small number of patients in the different sub-populations, we could not identify significant differences in the clinical outcome and therefore no associations with microbiota changes were searched. The use of bacterial lysates might play a role in microbiota rearrangement, but further data and advanced analysis are needed to prove this in less heterogeneous populations with higher numbers of samples considering the multiple influencing factors such as delivery method, age, environment, diet, antibiotic use, and type of infections to ultimately show any associations with prevention of rRTIs.

## 1 Introduction

Respiratory tract infections (RTIs) tend to recur in the pediatric population and, in the first years of life, are associated with increased risk of wheezing illness and asthma (Zomer–Kooijker et al., 2014). The overuse of antibiotics to treat acute RTIs contributes to the increase in the rate of antimicrobial resistance and related lack of efficacy as well as to the disruption of the host microbiota, which is essential for immune homeostasis. Both effects induce a self-perpetuating vicious cycle of infection–inflammation–reinfection that potentially leads to chronic respiratory conditions (Dethlefsen et al., 2008). Because of the above, solutions to address poor respiratory health in young children should be sought around pathogen exposure, commensal colonization, and immune training. Based on the “hygiene hypothesis”, the “farm-dust” effect, and the more recent concept of “innate immune training” (Ober et al., 2017; Netea et al., 2020), it has been suggested that the exposure to some microorganisms or microbial-derived components administered orally might influence the development and functions of gut and, secondarily, airway microbiota, mimicking the protective natural exposure that is needed for a healthy respiratory system (Esposito et al., 2018; Rossi et al., 2020). Oral bacterial lysates have been shown to act as immunomodulators able to shape the immune response to protect children from RTIs and associated wheezing (Esposito et al., 2018). An interesting hypothesis is that oral administration of whole bacteria or bacterial component can lead to a change in the gut microbiota composition by colonization and/or outgrowth of “good” strains. This could influence the composition of the airway microbiota either indirectly, by the migration of bacterial components or metabolites to the lungs to favor the outgrowth of “good” bacteria, or directly *via* microaspiration of these from the gastroesophageal tract to the airways. These may lead to the re–establishment of a health–promoting microbiota and have some therapeutic effects (Gollwitzer and Marsland, 2014). As such, an oral respiratory inactivate pathogenic bacterial lysate (manufacturing code name OM–85) could exert its effects by creating the conditions within the mucosal microbiome interface for the growth of beneficial bacteria or for limiting their outgrowth or repletion. This might, in turn, have a positive regulatory effect on airway inflammation such as shown in animal models of viral/bacterial superinfections and asthma (Karimi et al., 2009). The efficacy and safety of OM–85 was investigated in the OM–85–Pediatric rRTIs (OMPeR) study (EudraCT: 2016–002705–19), conducted in pre–school children (*n* = 288, age 1 to 6 years) with a history of recurrent RTIs (rRTIs) (Esposito et al., 2019). RTIs were significantly lower among patients receiving the standard regimen of OM–85 than among those given placebo (33% vs. 65.1%, *p* < 0.0001). OM–85 is an oral extract of bacterial lysates of 21 strains of eight known respiratory pathogens, *Haemophilus influenzae*, *Streptococcus pneumoniae*, *Klebsiella pneumoniae* subsp. *pneumoniae* and subsp. *ozaena*, *Staphylococcus aureus*, *Streptococcus pyogenes*, *Streptococcus viridans*, and *Moraxella catarrhalis*. The mechanism of action of OM–85 has been deeply reviewed and the main immunological effects have been described, nevertheless, the effect of this lysate on microbiota composition in children with rRTIs remained to be unraveled (Rossi et al., 2019). Therefore, a description of both gut and nasopharynx (NP) microbial composition in this population of children was performed considering common influencing factors reported in the OMPeR study demographics, e.g., age, atopy, and number of RTIs and antibiotics. A particular attention was given to age because children <2 years of age represent a window of opportunity to manipulate the microbiome and, as pointed out by Thomas et al., after 3 years of life, the gut microbiota is relatively more stable (Thomas et al., 2015). Furthermore, an exploratory analysis aimed at identifying any sign of possible microbiota rearrangement associated with the prophylactic use of OM–85 has been conducted. To our knowledge, this is the first study describing microbiota profiles in children with rRTIs receiving an oral bacterial lysate.

## 2 Materials and methods

### 2.1 Patient population

Children included in the clinical study OMPeR underwent biological sampling for both the NP and gut microbiome analysis. OMPeR was a phase IV, randomized, double–blind, placebo–controlled, single–center trial, which enrolled 288 patients and aimed at assessing the efficacy and safety of the oral bacterial lysates OM–85 reducing acute RTIs in pre–school children affected by rRTI defined as at least six acute episodes in the previous year. The active immunotherapy was administered as either 3.5 mg of OM–85 once a day for the first 10 days of the first 3 months of the 6–month study (group A) or once a day for the first 10 days of the 6–month study (group C). Matching placebo was administered to keep the double–blind condition (group B). The randomization 3:3:1 (OM–85, 10 days for 3 months, placebo or OM–85, 10 days for 6 months) was done at the beginning of the infective season (September/October), and the three groups were observed for 6 months. Children with malformations of the cardiovascular system and the respiratory tract, with chronic lung, kidney, or liver diseases, with primary or secondary immunodeficiency, and with cancer, malnutrition, and severe allergic manifestations such as atopic dermatitis and asthma were excluded. In addition, patients who received antibiotics and systemic, inhaled, or oral steroids within 4 weeks before enrollment were not included in the clinical trial and therefore excluded from our microbiome analysis.

### 2.2 Biological samples collection, processing, and DNA extraction

Both stools and NP swabs were collected at visit 2 (day −1, before the randomization) as baseline, and at V5 (month 6, end of the study) for microbiome essays. Stools were collected by the parents at home, as immediate freezing of fresh sample at -80°C was not possible, the specimens were stored at +4°C in anaerobic atmosphere for a short period (up to 24 h) and then frozen at −80°C in the microbiology laboratory of University of Milan, Pediatrics Clinic. In the same laboratory, NP swabs were obtained. NP samples were collected by trained personnel using sterile dry cotton–headed swabs (MASTASWAB MD 559, MAST Diagnostica GmbH, Reinfeld, Germany) by five circular rubbings about 1 cm from the nares. Secretion and other material were collected from the soft and moving part of the nose. After sampling, the swabs were immediately placed back in the collection tube and stored within 24 h at −80°C. The samples were processed using the kit for DNA isolation MoBio PowerLyzer, PowerSoil DNA isolation kit (Mobio, Loker Ave West, Carlsbad, CA, USA) according to the manufacturer protocol for extraction. A fecal sample of 200 μg was used, suspended in 200 μl of sterile water. The specimen was shaken first using the TISSUE LYZER 30–Hz impulses for 10 s for a total duration of 2 min. The sample was then centrifuged at 13,000*g* for 10 min and the supernatant was discharged. The pellet was transferred into a GLASS BEAD TUBE together with 750 μl of buffer Bead sol. The sample was warmed up first for 10 min at 65°C, then for 10 min more but at a temperature of 95°C. After adding 60 μl of C1 buffer, the sample underwent the proper lytic process by TISSUE LYZER 30–Hz impulses for 5 min + 5 min short bead–beating time, for a total of 10 min. The DNA material extractions were followed by a purification step to avoid that some constituents (e.g., scatols, fecols, and other aromatic acids) might inhibit the sequencing reactions. The commercial sequencing platform kit used for library generation (16S Illumina) included reagents to bind and remove known inhibitory compounds (Terranova et al., 2018). The NP swab samples underwent the processing and extraction process described by the same manufacturer (Mobio, Loker Ave West, Carlsbad, CA, USA). In our lab, the swabs were placed in the extraction tube with 750 μl of Bead sol buffer, and the tube was vortexed for 20 s. The sample could be then temporarily stored at −20°C until further processing (Depner et al., 2017). The sample has been transferred into the GLAS BEAD TUBE with 60 μl of C1 buffer and it underwent the lytic process by TISSUE LYZER 30 Hz impulses for 5 min + 5 min short bead–beating time, for a total of 10 min. Following extraction, DNA was quantified by Qubit^®^ dsDNA HS Assay Kits (Thermo Fisher Scientific Inc., Massachusetts, USA).

### 2.3 DNA sequencing

In our laboratory, we followed the 16S Illumina MiSeq system protocol (https://support.illumina.com/documents/documentation/chemistry_documentation/16s/16s–metagenomic–library–prep–guide–15044223–b.pdf). Briefly, V3–V4 hypervariable regions of the 16S rRNA gene were amplified with 16S Amplicon PCR Forward Primer = 5’ TCGTCGGCAGCGTCAGATGTGTATAAGAGACAGCCTACGGG–NGGCWGCAG and 16S Amplicon PCR Reverse Primer = 5’ GTCTCGTGGGCTCGGA–GATGTGTATAAGAGACAGGACTACHVGGGTATCTAATCC. Libraries were generated by dual indexing strategy using the Nextera XT v2 Index Kit (Illumina, California, USA). PCR products were cleaned up using Agencourt AMPure XP beads (Beckman Coulter Genomics, Minnesota, USA) and DNA quantification and quality were assessed by Qubit^®^ dsDNA HS Assay Kits and Bioanalyzer (Agilent Technology, California, USA), respectively. Equimolar DNA amounts from each sample library were pooled together. Finally, the pool was sequenced on the MiSeq sequencing instrument using a 2 × 250 cycle MiSeq Reagent Kit v2 (Illumina, California, USA). Reagent blank samples were subjected to all the steps of library preparation, from DNA extraction to amplification, to avoid contamination bias. Bioanalyzer assay showed no amplification product in the blank samples. During the DNA processing of the raw sequence, data obtained contained sequences corresponding to sequencing adaptors and primers used for amplification, as the first step, these segments were trimmed away. Sequence data with a minimum length of 250 base pairs were processed and analyzed. Quality–control filters were used to identify such poor–quality reads and purge these from the data. Only reads with an average quality score of 30 or above (which represents an expected error rate of fewer than 1 base for every 1,000 bases) were selected for further analysis. Samples were rarefied to a read depth of 6,700 to ensure that a reasonable number of sequence reads have been obtained for each Operational Taxonomic Units (OTU). We applied the following standards: sequences with >97% of nucleotide identity were assumed to correspond to one or few correlated microorganisms (e.g., species) while a lower level of identity of >95% was used to identify sequence clusters of the same genus. These clusters (OTU) were classified by phylogeny using the classifier from the Greengenes database (https://greengenes.secondgenome.com/).

### 2.4 Microbiome library

The first step in our microbiome analysis was the elimination of data background noise. We used a 10% cutoff. Bacterial taxa that were present in a few specimens of the same group (<10%) or that were present in very low concentration once the specimens are put all together (i.e., for very few reads, e.g., <0.005% of reads in all) were discharged. The second step was to create the library for each specimen at T0 and T1, to make intra–group comparisons at different time points. For fecal samples, we considered the taxa being represented by at least 2% of all reads per sample. For the NP samples, we kept the taxa being represented by at least 1% of all reads per sample (Sarangi et al., 2019). QIIME Software was used for the initial analysis (Caporaso et al., 2010).

### 2.5 Data analysis

For this exploratory and descriptive analysis, we chose to present data as the relative abundance of major taxa. Descriptive statistics were reported in terms of means with standard deviations (SD) for quantitative data and in terms of absolute frequencies or percentages for qualitative data. Non–parametric Wilcoxon’s signed–rank test for paired quantitative data was used to compare intra–group data, i.e., between two different time points: at visit 2 (day −1, before the randomization) as baseline, and at visit 5 (month 6) at the end of the study. Unpaired Mann–Whitney U test was used to compare quantitative data in two different groups, i.e., age groups. For comparing mean age among the three treatment groups (i.e., OM–85/placebo, OM–85, placebo only), Kruskal–Wallis test followed by Bonferroni *post–hoc* test was used. For comparing qualitative data, Chi–square test or Fisher’s exact test in the case of expected frequencies < 5 was used.

## 3 Results

**Figure 1:**
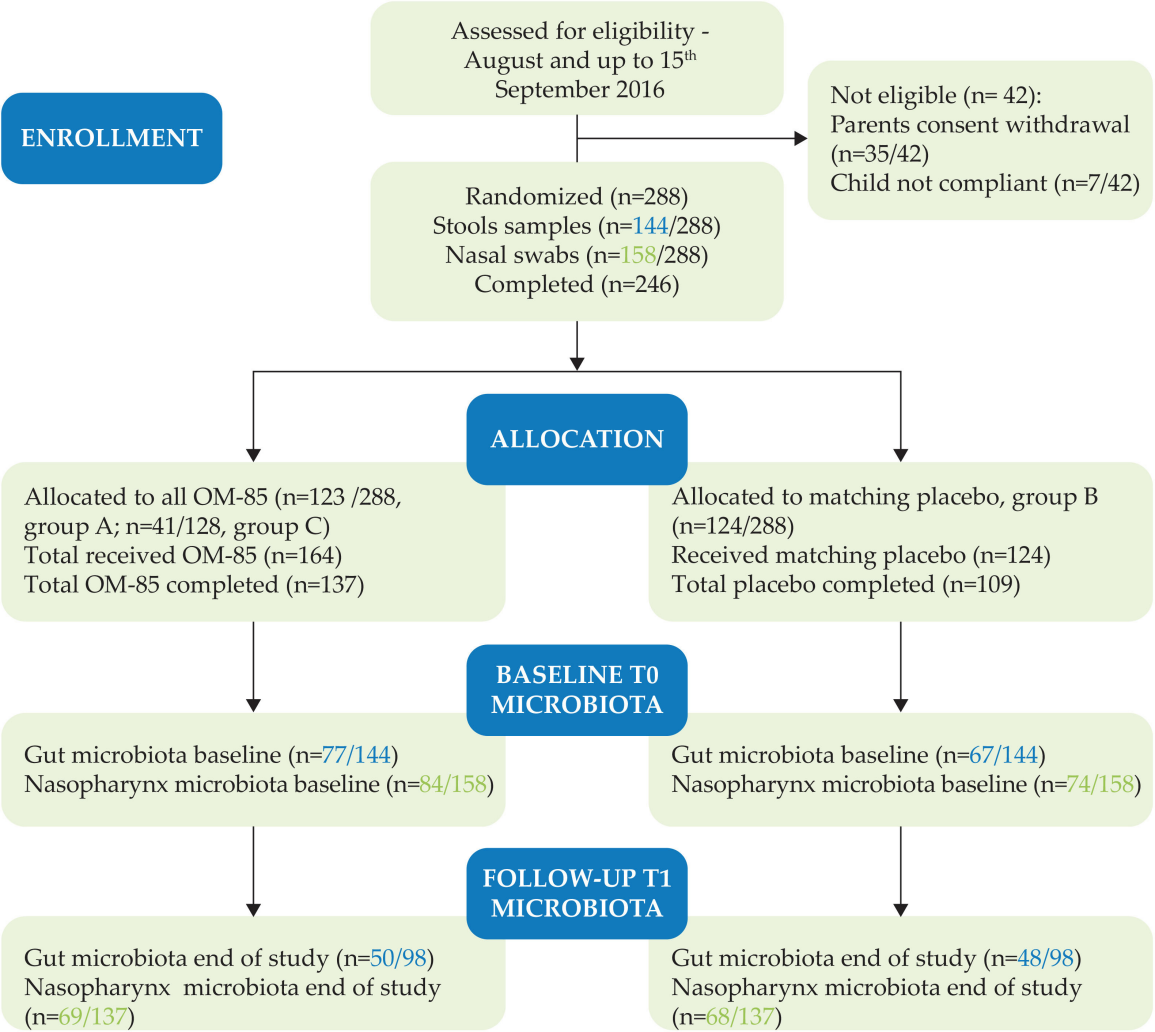
CONSORT. Number of gut and nasopharyngeal (NP) samples per treatment allocation (different active treatment groups A and C with OM–85 and group B with placebo) collected and analyzed at T0 and T1.

Out of the 288 children recruited in the OMPeR study, we could only collect and analyze 144 stool and 158 NP swabs samples at baseline (T0), approximately in half of the whole population. At the end of the study (T1), microbiota collection and analysis were performed in stool samples from 98 out of 144 patients and in 137 NP samples out of 158 patients who provided biological specimens at T0. Samples available at both T0 and T1 for each patient were used for longitudinal studies on gut (*n* = 98) and NP microbiota (*n* = 137) (**Figure 1**).

### 3.1 Age distribution in the study population and in the microbiota study subgroups at T0

Because of the strong influence on the microbiota composition, the age distribution across the studied groups was first verified. As reported in the demographic table of the OMPeR original article (Esposito et al., 2019), the three treatment groups (A, B, and C) were homogeneous for mean age in years ± SD at admission (3.6 ± 1.6, 3.7 ± 1.5, and 3.8 ± 1.7, respectively). In contrast, when looking at the 144 patients with gut microbiota profile available at T0, we found that the mean age of children receiving OM–85 treatment (A and C) was significantly lower compared to the placebo group (B) (*p* = 0.014) (**Table 1**). The difference was still significant when the data from active groups (A and C) were combined (*p* = 0.004) (**Table 2**), or when data related to infants and pre–school children were analyzed separately (*p* = 0.041) (**Table 3**). Similar findings were reported when analyzing the mean age and the distribution by the same age cutoff (infants and pre–schoolers) in the 158 children allocated to OM–85 and placebo groups for which NP microbiota analysis was available at T0 (data not shown).

**Table 1:** Mean (SD) age in years at T0 per randomization group (A, OM–85/placebo, B, placebo, C, OM–85) of all patients with available stool specimens and gut microbiota analysis (*n* = 144).

OM–85/Placebo (A) (*n* = 62)	Placebo (B) (*n* = 67)	OM–85 (C) (*n* = 15)	*p*–value
3.2 (1.6)	3.9 (1.6)	2.9 (1.4)	0.014

**Table 2:** Mean (SD) age in years at T0 per randomization groups receiving active prophylactic treatment (A, OM–85/placebo and C, OM–85) or placebo (B) of all patients with stool specimens and gut microbiota analysis available (*n* = 144).

All OM–85 (A + C) (*n* = 77)	Placebo (B) (*n* = 67)	*p*–value
3.1 (1.5)	3.9 (1.6)	0.004

**Table 3:** Number and percentage of patients <2 or ≥2 years of age with stool samples and gut microbiota analysis done at T0 (*n* = 144).

Age cutoff	All active (A + C) (*n* = 77)	Placebo (B) (*n* = 67)	*p*–value
<2 years [*n* (%)]	21 (27.3)	9 (13.4)	0.041
≥2 years [*n* (%)]	56 (72.7)	58 (86.6)	

### 3.2 Microbiota profiling at T0

#### 3.2.1 Gut microbiota profiling at T0 in the total population and in the different age subgroups

**Figure 2:**
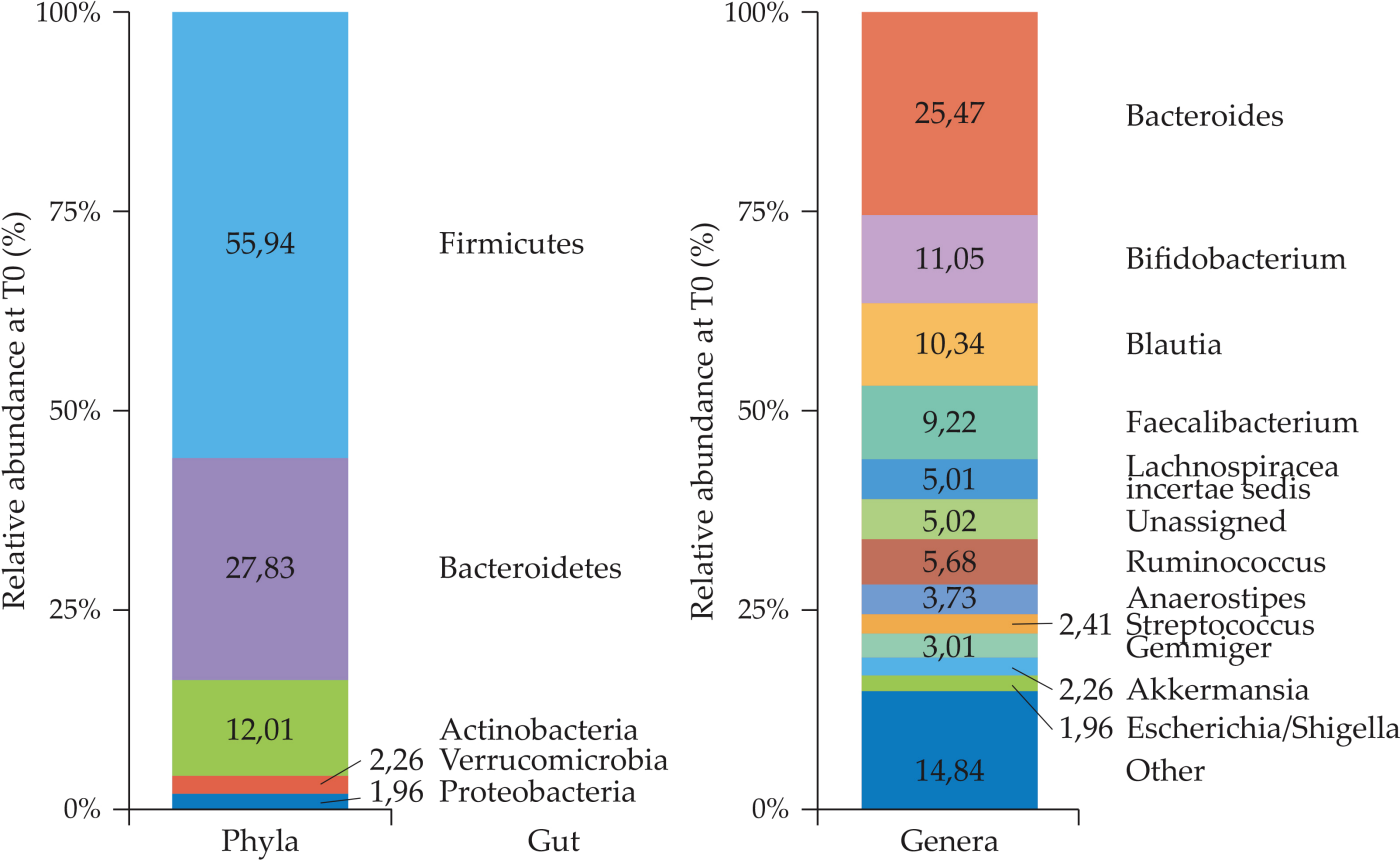
Gut microbiota profiles (phyla and genera) in the total population (*n* = 144) at T0.

**Figure 3:**
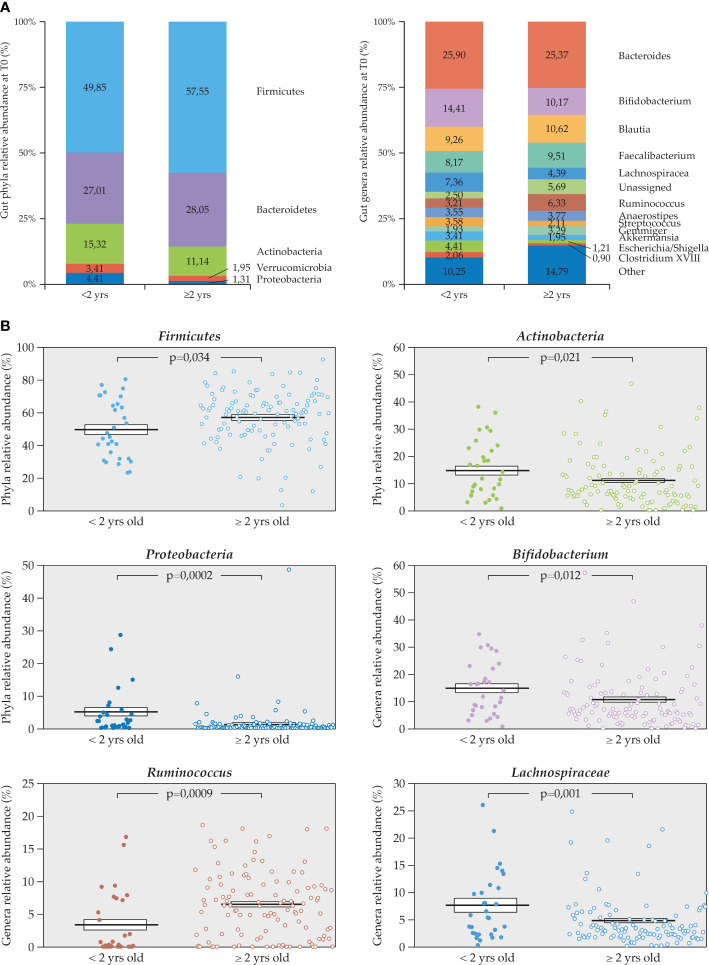
Relative percentage abundance of phyla and genera in the gut microbiota at T0. **(A)** Comparison between <2 (*n* = 30) and ≥2 years of age (*n* = 114) subgroups, **(B)** statistically significant differences in relative abundance of specific taxa, *Firmicutes, Proteobacteria*, and *Actinobacteria* (phyla) and *Bifidobacterium, Ruminococcus*, and *Lachnospiraceae* (genera) in the two age subgroups (Mann U Whitney test).

The gut microbiota analysis at T0 was performed in the total population (*n* = 144), and in the <2 (*n* = 30) and ≥2 years of age (*n* = 114) subgroups, regardless of the treatment allocations, as reported in **Figures 2** and **3**, respectively.

The composition and relative percentage abundance of phyla in the gut were comparable with the one described in the literature for healthy children with *Firmicutes* being predominant followed by *Bacteroidetes* (**Figure 2**).

In children <2 years of age, *Firmicutes* were less abundant compared to what was observed in older children, while *vice versa*, *Actinobacteria* and *Proteobacteria* were relatively more abundant. Among genera, *Bifidobacterium* and *Lachnospiraceae* were more abundant in children <2 years of age while *Ruminococcus* was less abundant (**Figures 3A, B**). Other genera differed significantly such as *Veillonella* and *Dorea* (data not shown because of their very low relative abundance).

#### 3.2.2 NP microbiota at baseline in the total population and in the different age subgroups

**Figure 4:**
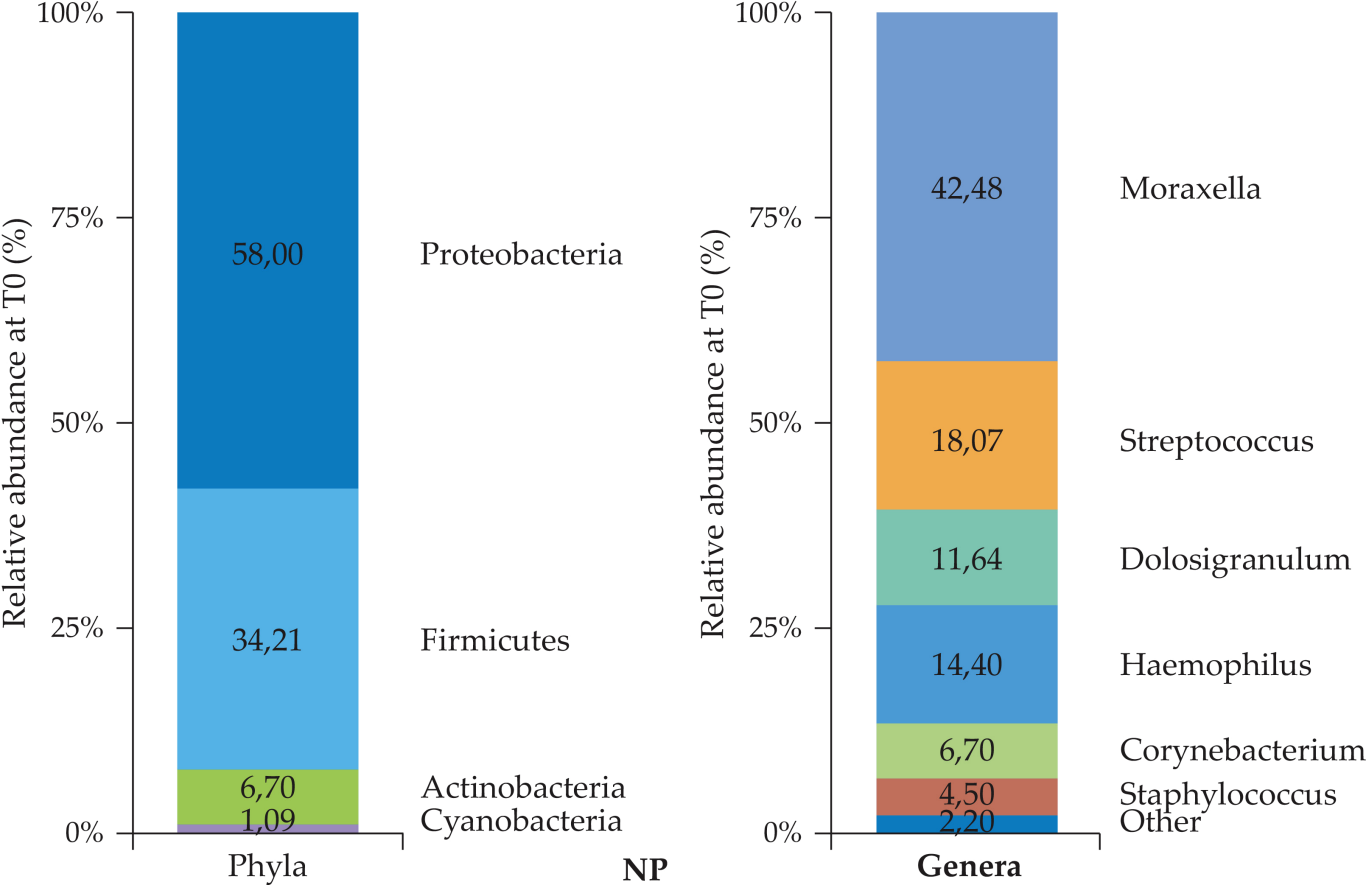
NP microbiota profiles (phyla and genera) in the total “NP sample” (*n* = 158) at T0.

The baseline NP microbial profiles were analyzed first in the total population (*n* = 158), and phyla and genera are reported in **Figure 4**.

The relative abundance of phyla was in line with what was described in the literature in children with highest abundance of *Proteobacteria* and *Firmicutes* followed by *Actinobacteria*. The relative abundance of genera was also reported at T0. The profiles in this case were not statistically different in the different age subgroups (data not shown).

#### 3.2.3 Distribution of gut microbiota taxa at T0 by other influencing factors (atopy and prior RTIs)

In the total patient group (*n* = 144), the baseline gut microbiota profiles were described and evaluated according to the presence or not of atopy and to the number (<3 or ≥3) of RTI episodes over 6 months prior to study entry. The number of atopic children with stool specimen was small (*n* = 31) compared to the non–atopic ones (*n* = 113). The mean age in years was higher in atopic children (4.11 ± 1.5) as compared with the non–atopic ones (3.28 ± 1.56, *p* = 0.006). The numbers of children who experienced <3 or ≥3 RTI episodes were respectively 49 and 95 and their mean ages in years were 3.63 ± 1.73 and 3.37 ± 1.5 (*p* = 0.43). Because of the very small numbers, we could not show any statistically significant difference in the gut microbial profiles at T0 between these patient subgroups. Furthermore, other influencing factors, such as the type of infections or the use and type of antibiotics prior to the study could not be analyzed because such historical data were not available.

#### 3.2.4 Distribution of NP microbiota taxa at T0 by other influencing factors (atopy and prior RTIs)

The baseline profiles were evaluated also for the NP microbiota in the total patient group (*n* = 158) and described according to the presence of atopy and to the number of RTI episodes. The mean age in years for the atopic (*n* = 30) and the non–atopic (*n* = 128) patients was statistically different, being 4.23 ± 1.5 and 3.28 ± 1.56, respectively (*p* = 0.0023). As described for the gut microbiota relative abundance, we could not observe statistically significant differences in the microbiota relative abundance by atopy.

**Figure 5:**
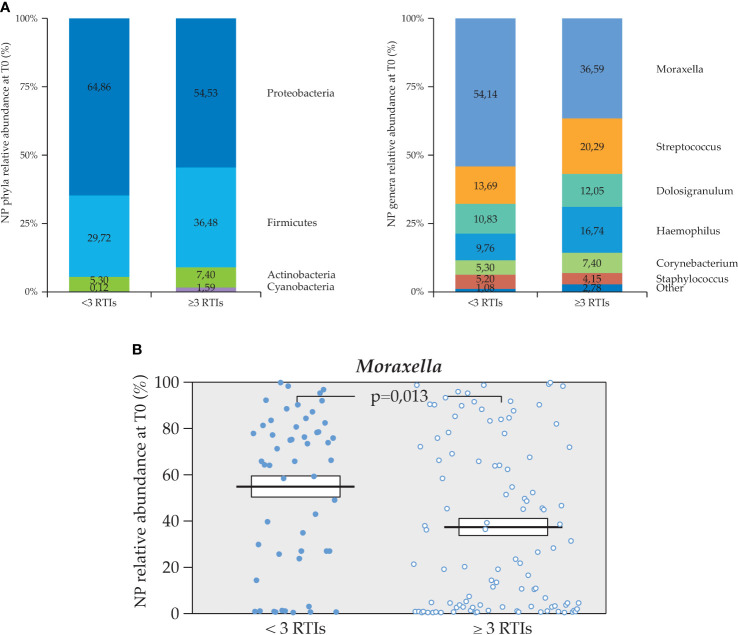
Relative percentage abundance of phyla and genera in the NP microbiota at T0. **(A)** Differences of NP microbiota profiles per number of RTIs in the 6 months prior to the study (n=53 <3 and n=104 ≥3 RTIs), **(B)** Statistically significant difference in relative abundance of *Moraxella* spp. between the two subgroups (Mann U Whitney test).

The number of rRTIs prior to the study was available for 157 out of 158 patients. The mean age was similar in the two groups, i.e., the 53 patients with <3 RTIs and the 104 patients with ≥3 RTIs. Some differences in the relative abundance of each taxon were observed (**Figure 5A**), but only *Moraxella* was significantly more enriched in the patients that had experienced <3 RTIs in the previous 6 months before the study entry (**Figure 5B**).

### 3.3 Gut microbiota profiling changes at T1

#### 3.3.1 Intra–group changes of gut microbiota profiles over the study (T1–T0)

**Figure 6:**
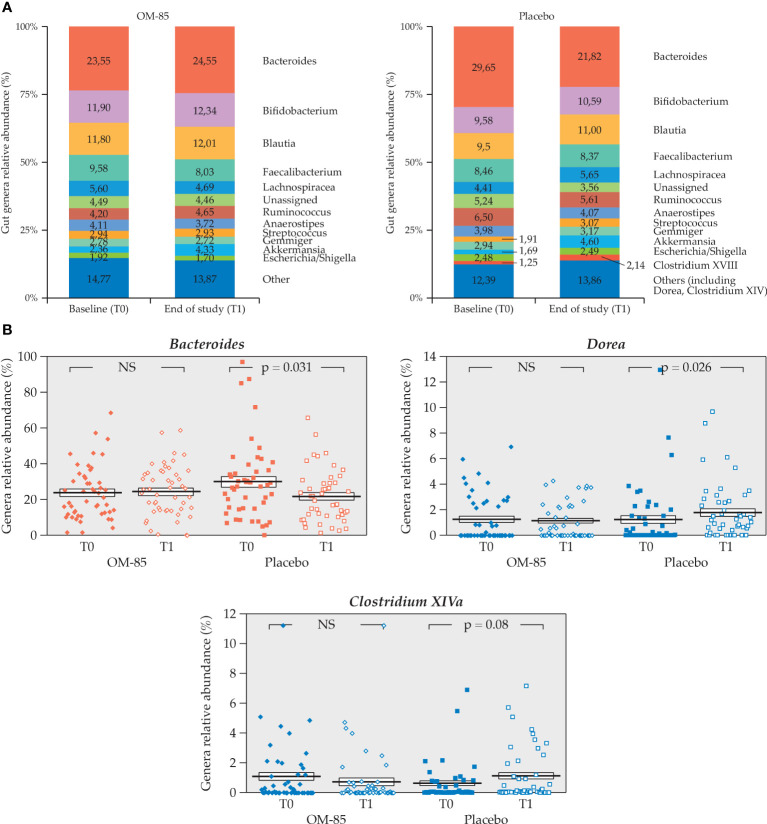
Intra–group change of gut microbiota relative abundance in both OM–85 and placebo groups (n=98). **(A)** Gut microbiota profiles at T0 and T1 for both treatment groups (n=50, OM-85 and n=48, placebo). **(B)** Statistically significant intra–group differences for genera *Bacteroides, Dorea*, and *Clostridium XIVa* in placebo (Wilcoxon’s signed–rank test). NS: not significant.

For such longitudinal study, only 98 gut samples were available (n=50 OM-85 and n=48 in placebo groups). Some statistically significant differences in relative abundance of specific taxa were observed, when comparing the two time points (**Figure 6A**). While the relative abundance of *Bacteroides* genus did not change over time in the OM–85 group, it decreased significantly in the placebo group (*p* = 0.03). In contrast, while no changes were observed for the *Clostridium XIVa* and *Dorea* in the OM–85 group, the relative abundance was increased with borderline significance for *Clostridium XIVa* and in a statistically significant manner for *Dorea* (*p* = 0.026) in the placebo group (**Figure 6B**).

#### 3.3.2 Intra–group changes of gut microbiota profiles over the study (T1–T0) in the different age subgroups

**Figure 7:**
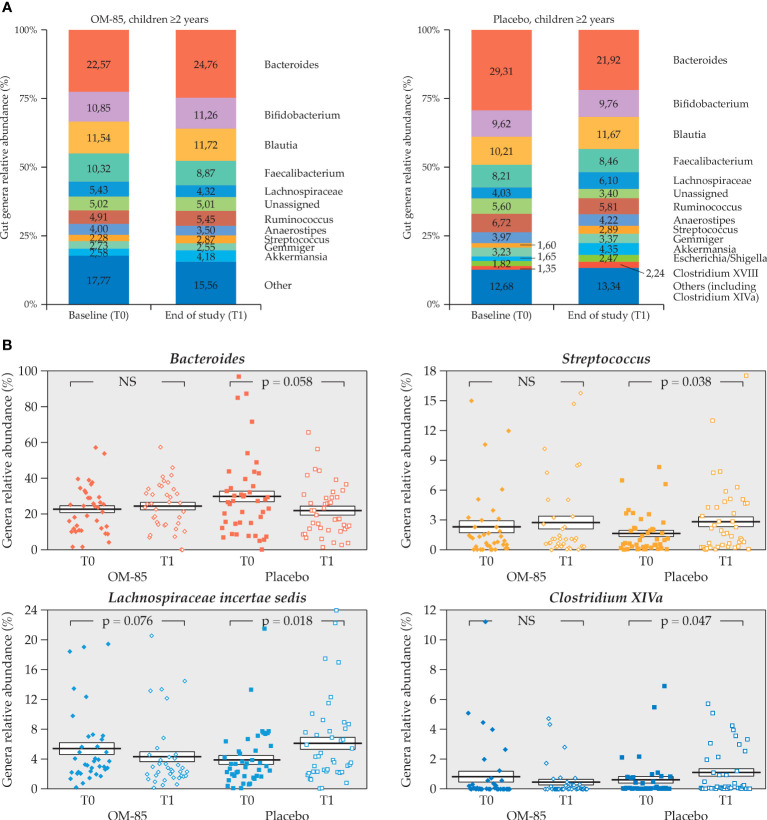
Intra–group change of gut microbiota relative abundance in both OM–85 and placebo group in the subgroup of children aged ≥2 years (n=80) **(A)** Gut microbiota profiles at T0 and T1 for both treatment groups (n=37, OM-85 and n=45, placebo). **(B)** Statistically significant intra–group differences in relative abundance of *Bacteroides, Streptococcus, Lachnospiraceae_incertae_sedis*, and *Clostridium XIVa* genera in placebo. (Wilcoxon’s signed–rank test). NS: not significant.

The intra–group comparisons of gut microbiota relative abundance in children <2 and ≥2 years of age were also made. Only 18 children <2 years of age had gut microbiota profiles at T0 and T1, and of these, 13 were in the OM–85 and only 5 were in the placebo subgroup. No statistically significant differences could be seen in this small subgroup of patients (data not shown). Differences were observed instead in the ≥2 years of age subgroup of children (*n* = 80), of whom 37 were in the OM–85 group and 43 were in the placebo group (**Figure 7A**). However, statistically significant differences were detected only in the placebo subgroup, with relative abundance reduction of *Bacteroides* and increase in *Streptococcus, Lachnospiraceae_incertae_sedis*, and *Clostridium XIVa* (**Figure 7B**). The gut microbiota remained more stable in this longitudinal study over the 6 months in the OM–85 subgroup.

### 3.4 OM–85 and placebo clinical response in the gut microbiota group

**Figure 8:**
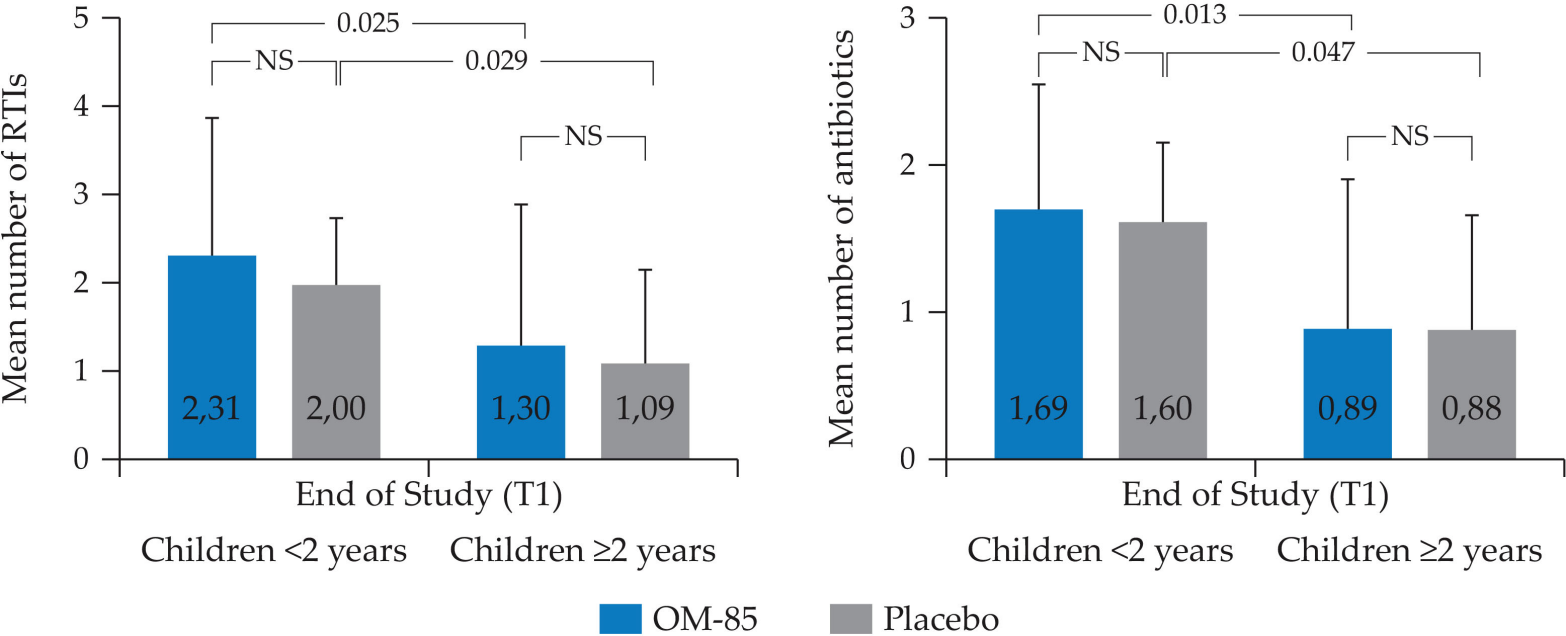
Mean number of RTIs and antibiotics used during the study and recorded at T1 in the children (n=98), <2 (n=18) and ≥2 (n=80) years of age (Mann *U* Whitney test). RTIs = LRTIs, URTIs, AOM, and otorrhea. URTIs: upper respiratory tract infections, LRTIs, lower respiratory tract infection; AOM, acute otitis media; NS, not significant.

In the 98 patients whose stool samples were collected for gut microbiota analysis both at T0 and at T1 (n=50 in OM–85 and n=48 in placebo group), no significant differences were observed in the mean number of RTIs at the end of the study (OM–85 = 1.56 ± 1.66, placebo = 1.19 ± 1.05, *p* = 0.62), as well as in the mean number of antibiotics (OM–85 = 1.10 ± 1.049, placebo 0.96 ± 0.80, *p* = 0.62) used during the study. The same analysis conducted using the age cutoff of 2 years showed a statistically significant higher mean number of RTIs and antibiotic use in the <2 years old subgroup at T1, as compared to the ≥ 2 years old subgroup (*p* = 0.025 and *p* = 0.013, respectively), but with no significant difference between OM–85 and placebo (**Figure 8**). No association between gut microbiota profiles and clinical response could be made. It should be noted that the mean number of antibiotics used over the study was very low, as the majority of RTIs were in the upper RT and did not require antibiotic prescription.

### 3.5 NP microbiota profiling changes at T1

#### 3.5.1 Intra–group changes of NP microbiota profiles over the study (T1–T0)

**Figure 9:**
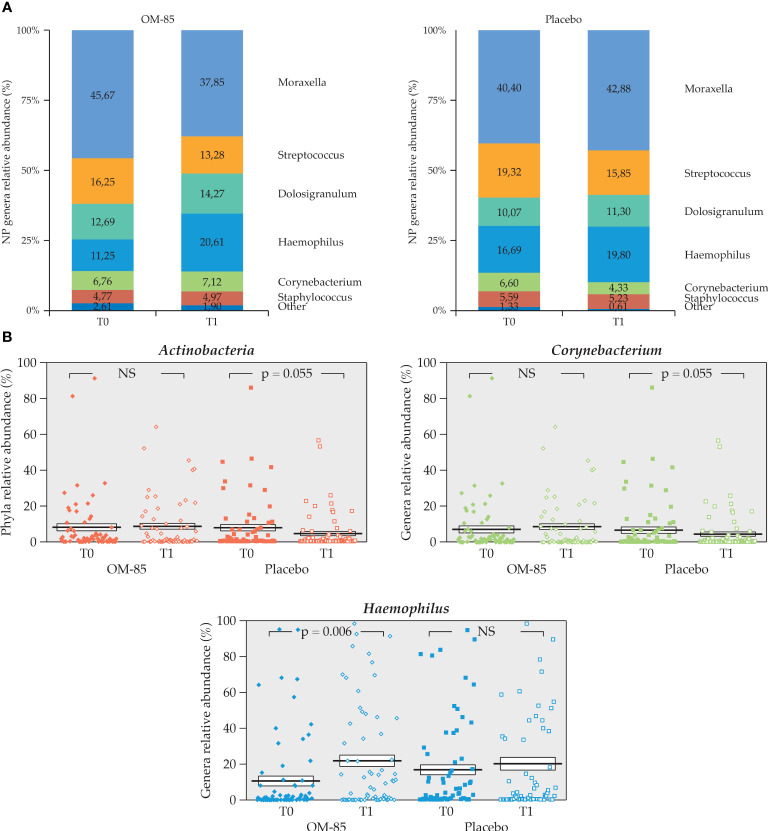
Intra–group change of NP microbiota composition in both OM–85 and placebo groups (n=137). **(A)** NP microbiota profiles at T0 and T1 for both treatment groups (n=69, OM-85 and n=68, placebo). **(B)** Statistically significant intra–group difference for *Actinobacteria, Corynebacterium* in placebo, and *Haemophilus* in OM-85 (Wilcoxon’s signed–rank test). NS: not significant.

Analysis of NP microbiota of the 137 patients (n=69 in OM-85 and n=68 in placebo groups) at T1 showed a significant decrease in the placebo group only of *Actinobacteria* phylum, an increase in relative abundance of *Haemophilus* in the OM–85 group, and a near to significant decrease of *Corynebacterium* in the placebo group compared to T0. Precisely, the *Actinobacteria* phylum decreased in placebo from 6.60% ± 1.63 to 4.33% ± 1.18 (*p* = 0.055). In addition, in the OM–85 group, the relative abundance of *Haemophilus* was 11.25% ± 22.47 at T0 and increased to 20.61% ± 22.55 at T1 (*p* = 0.006). In the placebo group, the relative abundance of *Corynebacterium* was 6.6% ± 13.45 at T0 and decreased to 4.33% ± 9.71 at T1 (*p* = 0.05) (**Figures 9A, B**).

#### 3.5.2 Intra–group changes of NP microbiota relative abundance over the study in the different age subgroups

**Figure 10:**
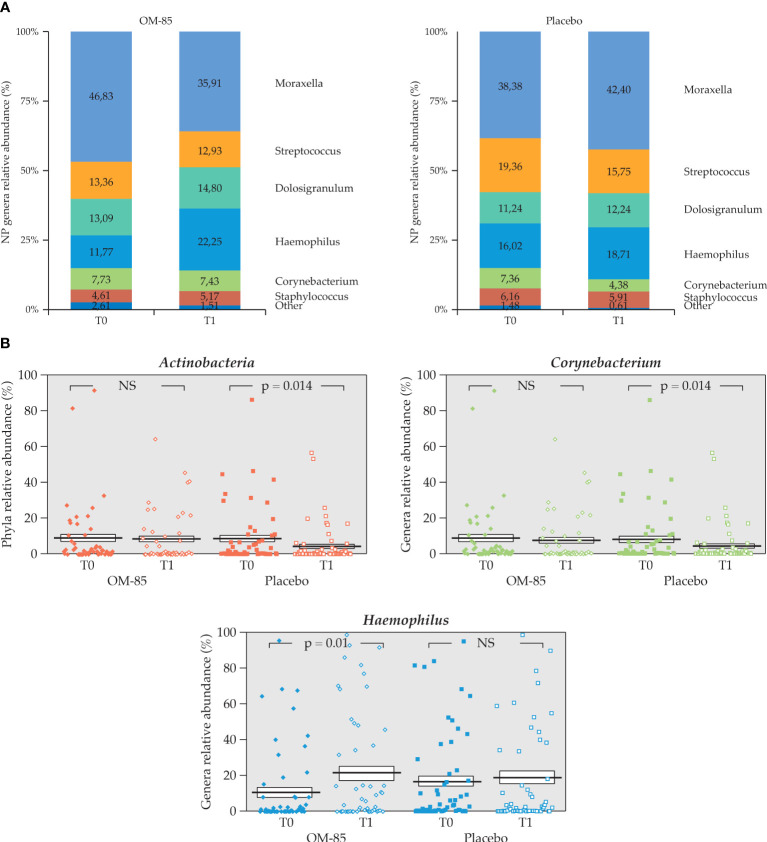
Intra–group change of NP microbiota relative abundance in OM–85 and placebo groups in the subgroup of children aged ≥2 years (*n* = 111). **(A)** NP microbiota profiles at T0 and T1 for both treatment groups (n=52, OM-85 and n=59, placebo). **(B)** Statistically significant intra–group difference in relative abundance for *Actinobacteria* phylum, and *Corynebacterium* in placebo and *Haemophilu*s genera in OM-85 (Wilcoxon’s signed–rank test). NS, not significant.

The mean age in years was not homogeneously distributed, with a lower mean age (*p* = 0.015) as well as a higher number of younger children included in the OM–85 group. We observed some differences in NP microbiota relative abundance at T1 only in children of ≥2 years (*n* = 111), in OM–85 (*n* = 52) and placebo groups (*n* = 59), both at the phylum level and the genus level. The *Actinobacteria* phylum did not change in the OM–85 group, but it decreased in the placebo group (7.36% ± 14.24 to 4.38% ± 10.08, *p* = 0.0136), and *Haemophilus* increased in the OM–85 group (11.77% ± 22.33 to 22.25% ± 31.08, *p* = 0.010) while the *Corynebacterium* decreased in the placebo group (7.36% ± 14.24 to 4.38 ± 10.08, *p* = 0.013), as shown in **Figures 10A, B**.

### 3.6 OM–85 and placebo clinical response in the NP microbiota group

No significant difference could be detected between the OM–85 and placebo group in the mean number of RTIs and the number of antibiotics use, during the study in patients with NP swabs both at T0 and at T1 (*n* = 137). However, when the different age groups were compared, a statistically significant higher mean number and frequency of RTIs and of antibiotic prescriptions were found in the youngest children, both in the OM–85 group and in the placebo group (**Table 4**). No association between NP microbiota profiles and clinical response could be made.

**Table 4:** Mean (SD) number of antibiotic prescriptions and respiratory infections, use of antibiotics (yes, no), and RTI occurrence (yes, no) in subgroups of children <2 years or ≥2 years during the study in the OM–85 and the placebo groups. .

	OM–85 (*n* = 69)		*p*–value		Placebo (*n* = 68)	*p*–value
	<2 years (*n* = 17)	≥2 years (*n* = 52)		<2 years (*n* = 9)	≥2 years (*n* = 59)	
Number of antibiotics	1.65 (0.79)	0.92 (0.97)	**0.005**	1.67 (0.71)	0.90 (0.78)	**0.010**
Number of RTIs^1^	2.29 (1.45)	1.29 (1.58)	**0.007**	1.78 (0.97)	1.09 (1.06)	**0.033**
						
Antibiotics: Yes (%)	16 (94.12%)	30 (57.69%)	**0.007 F**	9 (100%)	39 (66.1%)	**0.0495 F**
No (%)	1 (5.88%)	22 (42.31%)		0	20 (33.9%)	
RTI Yes (%)	16 (94.12%)	29 (55.77%)	**0.003 F**	8 (88.89%)	41 (69.49%)	0.43 F
No (%)	1 (5.88%)	23 (44.23%)		1 (11.11%)	18 (30.51%)	

^1^ URTIs, LRTIs, AOM, and otorrhea. URTIs: upper respiratory tract infections, LRTIs, lower respiratory tract infections; AOM, acute otitis media. The bold font means that the value is statistically significant.

## 4 Discussion

RTIs are common in the pediatrics population, especially in young children, because of the relative immaturity of the immune system and the microbiota, as well as the exposure to respiratory pathogens in childcare facilities and schools. Some children are more fragile than others, experiencing higher respiratory morbidity, characterized by RTI recurrence and/or more severe clinical manifestations. It is known that complex inter–talks between environmental and host factors such as immune components and microbiota metabolites move the needle towards a health or disease status in childhood (Man et al., 2017).

It has been hypothesized that reducing the risk for RTIs in infancy could be therapeutically achieved by accelerating early immune maturation/functional competence *via* enhancing the level of appropriate benign microbial–derived signaling to the developing innate immune system. The “immune training” led to a state of broad spectrum enhanced resistance to pathogens (Holt et al., 2019). In addition, a right balance between Th1/Th2 responses is key to avoid more severe LRTIs and sequelae. A few microbial–derived products have been shown to experimentally reproduce immune training effects. The bacterial lysate OM–85 has been extensively investigated, and it has shown to enhance deficient INF responses (Dang et al., 2017) to modulate the interplay between Th1 and Th2 mechanisms (Huber et al., 2005) and to potentially play a role in gut microbiota rearrangement (Rossi et al., 2019).

The aims of our work were to (a) describe microbiota profiles in rRTIs of pre–school children, starting with relative abundance, (b) describe microbiota profile changes associated with common influencing factors such as age, atopy, and number or RTIs, (c) describe any possible sign of effect of the oral bacterial lysate OM–85 when given as prophylaxis on microbiota relative abundance, and (d) make associations, if possible, with its clinical efficacy in prevention of rRTIs.

Performing the sub–analysis of the gut and NP microbiota derived from the pre–school children included in the OMPeR clinical study, we realized that the number of stool and NP swabs collected at T0 and analyzed by 16S rRNA sequencing was approximately half of the total enrolled population and that the number of stool samples as well as NP swabs available and analyzed at both T0 and T1 was smaller. This could negatively affect the possibility to reach the statistical significance, when evaluating the results of the study.

Furthermore, in contrast with what was reported in the OMPeR study original population, the demographic characteristics were not homogeneously distributed in the different treatment groups in our studied sub–population. There was a statistically significant difference in the mean age for children included in the “gut” and the “NP” samples groups at T0, with the youngest allocated to the OM–85 treated group, and this could be a bias to keep in mind when evaluating the results of our study. It is well known that the age plays a key role in the microbiota richness in children, indeed, the gut microbiota is relatively less stable (i.e., more influenced by factors such as breast feeding, diet, and past infections antibiotic use) below the 3 years of age (Thomas et al., 2015). Infancy (i.e., <2 years) might represent a better window of opportunity to manipulate the microbiota. Also, when considering the age cutoff of 2 years, a significant higher proportion of younger children was allocated to OM–85 compared to placebo. As expected, more significant differences in the relative abundance of phyla and genera were observed at T0 when comparing microbiota profiles by using the “clinical” age cutoff of 2 years. Therefore, we performed all the microbiota analysis in the total “gut” or “NP” samples, and in the subgroups of children <2 or ≥2 years to minimize the age bias. The sample size in these age subgroups was small, and this was particularly true for the youngest group, which was less represented in the OMPeR study population according to the eligibility criteria (age at enrollment from 1 to 6 years). This negatively affected the analysis especially in group comparisons.

We therefore decided to apply a stepwise approach to our microbiota analysis to minimize the costs. At T0, in the gut microbiota, the phylum *Firmicutes* was predominantly followed by *Bacteroidetes* and *Actinobacteria* phyla, as reported by other authors (Arumugam et al., 2011). In the NP microbiota, *Proteobacteria* and *Firmicutes* were more represented, followed by *Actinobacteria*. Also, these findings were in line with what was reported in the literature for this age group of children (Pichon et al., 2017). Our T0 findings confirmed that the gut microbiota composition is influenced by age, as shown by several statistically significant differences in the microbiota profiles of children <2 years vs. ≥2 years of age.

In NP microbiota analysis, at T0, we could identify only a significant difference in *Moraxella* spp., which was more abundant in children who had <3 RTIs during the study, this was independent of the age, because this was not significantly different in these two subgroups (<2 or ≥2 years). It is important to point out that rRTIs in children are usually defined as at least six to eight over a 12–month period. In the OMPeR study, pediatrics patients were recruited with a history of ≥6 RTIs in the previous year. As not all the episodes over the entire 12–month period prior to the study were recorded or dated for all the patients, we defined rRTIs instead by the number of three episodes over 6 months prior to study. We observed that a higher relative abundance of *Moraxella* spp. was associated with less recurrences. Other authors reported that *Moraxella* spp. might be associated with a healthier status in the elderly at risk for RTIs, while in the pediatric populations, the reports on this health–associated taxa (Man et al., 2020) are more conflicting as far as microbiota profile stability and association with RTIs (Bosch and McFall–Ngai, 2011; Biesbroek et al., 2014; Teo et al., 2015; Man et al., 2019). The differences in susceptibility to RTIs likely arise from the complex interplay between mucosa, innate and adaptive immunity, and airway microbiota (van den Munckhof et al., 2020).

Other factors might influence or are being associated with microbiota rearrangements in the pediatric population prone to RTIs, such as atopy and antibiotic use. The number of atopic children was overall smaller compared to the non–atopic ones. The reasons of such imbalance can be found in the exclusion of asthmatics from the study according to OMPeR eligibility criteria. Because of the above, we could not find any association of specific microbiota profiles with atopy. In addition, we could not perform other subgroup analysis by other influencing factors such as the type of infection and of antibiotic use because this kind of anamnestic data was not available.

At T1, the gut microbiota relative abundance showed that *Bacteroides* spp. were significantly decreased while *Dorea* spp. increased compared to T0 in the placebo group only. *Bacteroides* spp. were also statistically decreased in the placebo subgroup of ≥2 years of age, which was the largest age subgroup. Other genera proved to be less stable in the placebo group compared to OM–85 (increased *Streptococcus, Lachnospiraceae_incertae_sedis*, and *Clostridium XIV*). Despite small numbers, the inter–group analysis in this subgroup still showed a statistically significant difference in the change for the *Bacteroides* (borderline significance) and for *Lachnospiraceae_incertae_sedis* spp.

*Bacteroides* is a Gram–negative, non–spore–forming, obligate anaerobic bacteria normally found in the human intestines, mouth, upper respiratory tract, and genital tract. *Bacteroides* expresses polysaccharide A, which can induce regulatory T–cell growth and cytokine expression that are protective against inflammation. A lower level of *Bacteroides* has been associated with some inflammatory diseases such as inflammatory bowel diseases (IBDs) (Zhou and Zhi, 2016). *Bacteroides* has been considered protective by some authors (Lazar et al., 2018), while early colonization of *Bacteroides fragilis* was associated with asthma risk at 3 years of age (Vael et al., 2008). *Bacteroides fragilis* and *Bacteroides uniformis* have been identified to exert anti–inflammatory effects in animal models, and they might be considered as the next generation of probiotics (O’Toole et al., 2017).

*Lachnospiraceae* belong to the core of gut microbiota, colonizing the intestinal lumen from birth and increasing in terms of species richness and their relative abundances during the host’s life. In contrast to *Bacteroides*, *Lachnospiraceae* are in greater abundance in the irritable bowel syndrome (IBS) clone library (Rinninella et al., 2019). *Lachnospiraceae* might influence healthy functions, although different genera and species of this family are increased in diseases. Indeed, some metabolic syndrome, obesity, diabetes, liver diseases, IBD, and chronic kidney disease are all inflammatory conditions involving this family (including *Lachnospiraceae_incertae_sedis* or *Blautia* spp.). In addition, they seemed to play a role in depressive syndromes and multiple sclerosis syndrome (Vacca et al., 2020).

Diet influences the microbiota in older children, and this factor was not controlled over the study. Furthermore, the role of gut microbiota specific taxa is still controversial in regard to health or disease status, and limited data are available for children with high risk for RTIs.

No direct associations between gut microbiota relative abundance changes at T1 and clinical response (RTIs and antibiotic use during the study) could be detected. This can be explained by a few factors, such as the small samples size for the microbiota OMPeR sub–analysis, the non–homogeneous age distribution in the treatment groups, and the small sample size when analyzing the age subgroups. These limitations can also explain the lack of significant differences for the clinical endpoints (RTIs and antibiotic use) between OM–85 and placebo in the sub–analysis, differences detected in the OMPeR total study population (Esposito et al., 2019). Furthermore, the mean number of RTIs and of antibiotic use was very low probably because the majority of RTIs were in the upper airways and did not require antibiotic prescription.

The NP microbiota relative abundance intra–group analysis at T1 confirmed a more stable microbiota for the OM–85 group compared to placebo. Some statistically significant changes were detected in the placebo group in both the total population and in the larger group of children ≥2 years of age where we observed a decrease for the *Actinobacteria* phylum and *Corynebacterium*. *Haemophilus* increased in the OM–85 group. *Actinobacteria* and *Corynebacterium* seem to be associated with respiratory health, while a relative high colonization of *Haemophilus* is associated with increase of asthma risk (Hufnagl et al., 2020).

As far as the clinical response, when comparing the frequency of RTIs and antibiotic use between OM–85 and placebo during the study, there were less children with RTI and antibiotics in the OM–85 group, but these findings were not statistically significant. Therefore, it was not possible to make associations between NP microbiota profiles and clinical outcomes.

The clinical efficacy of OM–85 treatment shown in the original OMPeR study was lost in our sub–analysis. This was in contrast with the one observed in the OMPeR total study population. Indeed, in OMPeR, there were statistically significant differences in favor of OM–85 for upper RTIs (i.e., common cold/viral pharyngitis) as well as for acute otitis media (AOM). Furthermore, the percentage of patients with recurrent upper RTIs and AOM favored OM–85. Precisely, the number of patients with recurrences was approximately 50% among children given placebo and only 21% among those treated with OM–85. The reduction of the URTIs and ear complications, the most frequent ones in the pre–school pediatric population, was associated with a general lower respiratory disease burden, measured as missed days of schools for children and of work for parents. These parameters were significantly reduced in the OM–85 compared to the placebo group. The reasons for such discrepancy between clinical outcomes in our sub–analysis and the OMPeR total population might be several. Surely, the loss of the effect of the randomization and the reduced sample size can be pointed out.

Our work presents some analysis limitations such as the lack of statistical methods controlling for multiple comparisons and multivariate analysis to assess sources of variation compared to the treatments. We did not apply statistical methods controlling for multiple comparisons because of the relatively small sample size of the subgroups, also considering the population heterogenicity. In addition, important microbiota influencing factors, such as method of delivery, gestational age, food source, and pets, were not assessed and reported in the OMPeR clinical trial, with the microbiota assessment only an ancillary study, and we could have not controlled for them. Furthermore, as far as the clinical endpoint, we could not confirm any significant difference between the subgroups, therefore, no further multivariate analysis to assess sources of variation compared to the treatments was done. The collaboration for metagenomic analysis with another research group on expected additional data will help better define the possible correlation between OM–85–induced changes in microbiome composition and clinical results in children with RTIs.

In conclusion, our study, which is registered in EudraCT: 2016–00705–19, is one of the few clinical trials assessing both gut and NP microbiota in pre–school children at risk for RTIs. In addition, it is, to our knowledge, the first study aimed at describing the microbiota relative abundance in patients treated with the oral bacterial lysate OM–85. Other studies used probiotic and prebiotic, mainly *Lactobacillus*, *Bifidobacterium*, and *Enterococcus*, to modulate gut microbiota, a promising approach against viral RTIs *via* host innate and adaptive immunity regulation (Shi et al., 2021).

Some authors are indeed suggesting that such kind of compounds themselves might mimic and even rearrange the gut and, indirectly, airway microbiota (Ober et al., 2017; Rossi et al., 2019). Others observed that bacterial–derived compounds might play a role in innate training, as it has been published for bacilli Calmette–Guerin (BCG) oral vaccine (Netea et al., 2020). Indeed, Mantovani et al. (Mantovani and Netea, 2020) suggested that the exposure not only to selected vaccines, but also to microbial components, can increase the baseline tone of innate immunity and trigger pathogen–agnostic resistance. Attention has converged on the importance of intervening at early life/stages, with the goal of reducing RTI severity and recurrences as well as preventing the progression to chronicity. To this end, the training of the immune system in early childhood represents an important strategy for preventing RTI–related morbidity and minimizing long–term consequences. Training the immune system with OM–85 might induce long–lasting changes in host microbiota and possibly in innate immunity, resulting in an enhanced response to infection by unrelated pathogens. Further studies are therefore needed in infancy, in a larger patient population, designed for metagenomics analysis and across more viral seasons to clarify its role in microbiota rearrangements.

## Data availability statement

The original contributions presented in the study are publicly available. This data can be found here: SRA; PRJNA488913. In addition, the data presented in the study are deposited in the European Clinical Trial registry (https://www.clinicaltrialsregister.eu/ctr-search/trial/2016-002705-19/IT)”, with access number 2016-002705-19.

## Ethics statement

The studies involving human participants were reviewed and approved by Fondazione IRCCS Ca’ Granda Ospedale Maggiore Policlinico, Milan, Italy. Written informed consent to participate in this study was provided by the participants’ legal guardian/next of kind.

## Author contributions

Conceptualization: SE and NP. Methodology: SE and NP. Software: SE. Validation: SE and NP. Formal analysis: SE, LR, and AA. Investigation: LR and AA. Resources: SE. Data curation and interpretation: SE, GR, and SB. Writing—original draft preparation: SB. Writing—review and editing: SB, SE, and GR. Visualization: SB. Supervision: SE and NP. Project administration: SE. Funding acquisition: SE. All authors have read and agreed to the published version of the manuscript. SB contributed to microbiota results interpretation and manuscript preparation as an Industrial International PhD student in “System Biology in Immunology and Infectious Pathologies”.

## Funding

This research was funded by WAidid, Word Association for Infectious Diseases and Immunological Disorders.

## Acknowledgments

The investigational medicinal product and placebo were supplied by OMPharma S.A.Meyrin, Switzerland) as an in–kind donation.

## Conflict of interest

The authors declare that the research was conducted in the absence of any commercial or financial relationships that could be construed as a potential conflict of interest.

## Publisher’s note

All claims expressed in this article are solely those of the authors and do not necessarily represent those of their affiliated organizations, or those of the publisher, the editors and the reviewers. Any product that may be evaluated in this article, or claim that may be made by its manufacturer, is not guaranteed or endorsed by the publisher.
